# Paracolic Gutter Abscess Associated with Bladder Diverticulum Following Proctectomy: A Case Report

**DOI:** 10.51894/001c.165985

**Published:** 2026-07-31

**Authors:** Elaine Ognjanovski, David I. LeRoy, Ermal Hasalliu, Darby Durrant, Cassie Konja

**Affiliations:** 1 Henry Ford - Warren

## 110

### Case Presentation

The patient is a 54-year-old male with a history of prostate adenocarcinoma and hypertension who underwent laparoscopic prostatectomy. Post-operative course was complicated by pelvic hematoma with concerns for active extravasation, managed conservatively. He developed multifocal pulmonary embolism and was started on Eliquis. Three weeks after discharge he presented with right-sided abdominal and rib pain. The pain began after Foley catheter removal and was described as constant and shooting in nature, only minimally relieved by outpatient analgesics.

He denied any trauma, chest pain, shortness of breath, gastrointestinal symptoms, or urinary symptoms. Initial laboratory evaluation showed leukocytosis, elevated alkaline phosphatase and lipase. Urinalysis was positive for a urinary tract infection (UTI). CT abdomen/pelvis with contrast revealed a new 14.7cm fluid collection in the right paracolic gutter concerning for abscess. Imaging also demonstrated a large urinary bladder diverticulum, bladder circumferential wall thickening concerning for inflammatory cystitis. Interventional Radiology was consulted for drain placement for patient’s right paracolic fluid collection and antibiotics were initiated. Foley catheter was placed by urology and patient was admitted to the intensive care unit for closer monitoring. Patient clinically improved after drain placement and was discharged home with close follow-up with urology.

### Discussion

The right paracolic gutter serves as an important passageway allowing infected fluid to track within the peritoneal cavity, predisposing to abscess formation. Paracolic gutter abscess formation is an uncommon post prostatectomy complication and is more frequently associated with intraabdominal infections such as diverticulosis. In this case, the presence of a bladder diverticulum, postoperative urinary retention, and inflammatory cystitis contributed to UTI with spread of infected fluid into the paracolic gutter. CT imaging played a critical role in identifying the abscess, as clinical presentation and lab findings alone were insufficient in leading to final diagnosis.

### Conclusion

This case highlights a rare postoperative complication in which a large paracolic gutter abscess developed in the presence of a bladder diverticulum. Extension into the paracolic gutter is uncommon and requires early detection due to nonspecific presentation. Maintaining a broad differential diagnosis when evaluating postoperative abdominal pain, especially in patients with structural urinary abnormalities is essential to prevent morbidity.

